# Exit from Naive Pluripotency Induces a Transient X Chromosome Inactivation-like State in Males

**DOI:** 10.1016/j.stem.2018.05.001

**Published:** 2018-06-01

**Authors:** Elsa J. Sousa, Hannah T. Stuart, Lawrence E. Bates, Mohammadmersad Ghorbani, Jennifer Nichols, Sabine Dietmann, José C.R. Silva

**Affiliations:** 1 Wellcome-MRC Cambridge Stem Cell Institute, University of Cambridge, Cambridge CB2 1QR, UK; 2 Graduate Program in Areas of Basic and Applied Biology, Instituto de Ciências Biomédicas Abel Salazar, Universidade do Porto, 4050-313 Porto, Portugal; 3 Department of Biochemistry, University of Cambridge, Cambridge CB2 1GA, UK; 4 Department of Physiology, Development and Neuroscience, University of Cambridge, Cambridge CB2 3EG, UK

## Abstract

A hallmark of naive pluripotency is the presence of two active X chromosomes in females. It is not clear whether prevention of X chromosome inactivation (XCI) is mediated by gene networks that preserve the naive state. Here, we show that robust naive pluripotent stem cell (nPSC) self-renewal represses expression of *Xist*, the master regulator of XCI. We found that nPSCs accumulate *Xist* on the male X chromosome and on both female X chromosomes as they become NANOG negative at the onset of differentiation. This is accompanied by the appearance of a repressive chromatin signature and partial X-linked gene silencing, suggesting a transient and rapid XCI-like state in male nPSCs. In the embryo, *Xist* is transiently expressed in males and in females from both X chromosomes at the onset of naive epiblast differentiation. In conclusion, we propose that XCI initiation is gender independent and triggered by destabilization of naive identity, suggesting that gender-specific mechanisms follow, rather than precede, XCI initiation.

## Highlights

• A robust naive pluripotent transcription factor network abolishes expression of Xist
• Xist accumulates on the male X and both female Xs at the onset of differentiation
• Males undergo transient and rapid partial XCI
• Male XCI is triggered by downregulation of the naive identity

## Graphical Abstract

**Figure:**
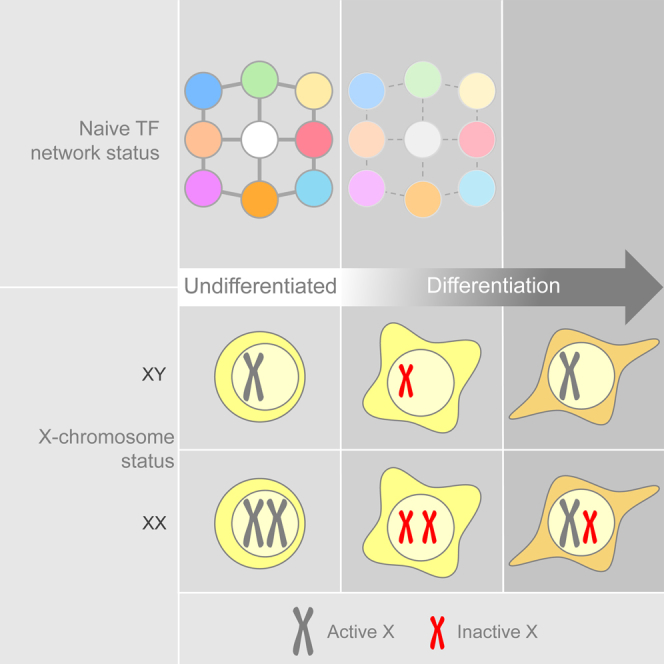

## Teaser

Silva and colleagues report that the initiation of X chromosome inactivation takes place in males and on both X chromosomes in females. This is transient and rapid and is triggered by downregulation of naive pluripotent transcription factors during the onset of differentiation.

## Introduction

In order to achieve dosage compensation, female mammals have one inactive X chromosome (Xi). However, in female murine embryos, the Xi is reactivated in the pre-implantation blastocyst (Mak et al., 2004, Okamoto et al., 2004) specifically in the cells of the naive pluripotent epiblast (Silva et al., 2009). Their *in vitro* counterpart, naive pluripotent stem cells (nPSCs), retain this embryonic feature, making them an excellent model system to study X chromosome inactivation (XCI). XCI is initiated upon differentiation of female nPSCs and is characterized by monoallelic upregulation of *Xist*, the non-coding RNA which coats the future Xi in *cis* (Panning et al., 1997, Sheardown et al., 1997). In contrast, *Xist* expression is extinguished during differentiation of male nPSCs.

The link between a naive pluripotent cellular identity and the lack of a Xi in females is still poorly understood. In the pre-implantation blastocyst, reactivation of the Xi occurs in cells expressing the nPSC marker NANOG (Silva et al., 2009). Moreover, NANOG and other members of the naive transcriptional network were found to bind to *Xist* intron 1 (Navarro et al., 2008). Deletion of *Nanog* and *Oct4* was shown to induce a moderate upregulation of *Xist* (Navarro et al., 2008), but deletion of *Xist* intron 1 was shown to be dispensable for XCI and did not affect *Xist* expression (Minkovsky et al., 2013).

X chromosome reactivation (XCR) is also a feature during *in vitro* nuclear reprogramming to naive pluripotent cell identity (Tada et al., 2001). The general consensus is that naive pluripotent gene regulators must play a role both *in vivo* and *in vitro* XCR (Navarro et al., 2008, Navarro et al., 2010, Navarro et al., 2011, Pasque and Plath, 2015, Pasque et al., 2014, Payer et al., 2013, Silva et al., 2009).

Studies investigating the process of XCI have largely been conducted *in vitro* and using nPSCs cultured in serum/LIF (SL) conditions. This is known to be suboptimal, as it induces transcriptional heterogeneity of pluripotency factors (Chambers et al., 2007), promotes an overall weak naive transcription factor (TF) network in which spontaneous differentiation and increased expression of lineage markers are observed (Marks et al., 2012), and exhibits epigenetic constraints (Ficz et al., 2013, Habibi et al., 2013, Leitch et al., 2013, Marks et al., 2012). It is also known to reduce reprogramming efficiency (Silva et al., 2008) and to decrease the ability of nPSCs to enter embryonic development (Alexandrova et al., 2016). Using defined serum-free medium containing LIF and inhibitors of mitogen-activated protein kinase signaling and glycogen synthase kinase-3β (2iL), these limitations have been overcome (Silva et al., 2008, Silva et al., 2009, Ying et al., 2008). 2iL acts on the TF network governing the naive identity by boosting its expression (Martello and Smith, 2014). In addition, nPSCs cultured in 2iL exhibit a transcriptional signature that is similar to the naive pluripotent epiblast (Boroviak et al., 2015). However, it is unknown whether increased transcriptional homogeneity and pluripotent TF robustness have an impact on the process of XCI.

Here, we assessed the relationship between naive pluripotent cell identity and the process of XCI. This uncovered unexpected XCI events during differentiation of both male and female nPSCs. These observations impact our understanding of XCI and its relationship with the naive pluripotent identity.

## Results

### Robust nPSC Self-Renewal Abolishes *Xist* Expression

**Figure 1:**
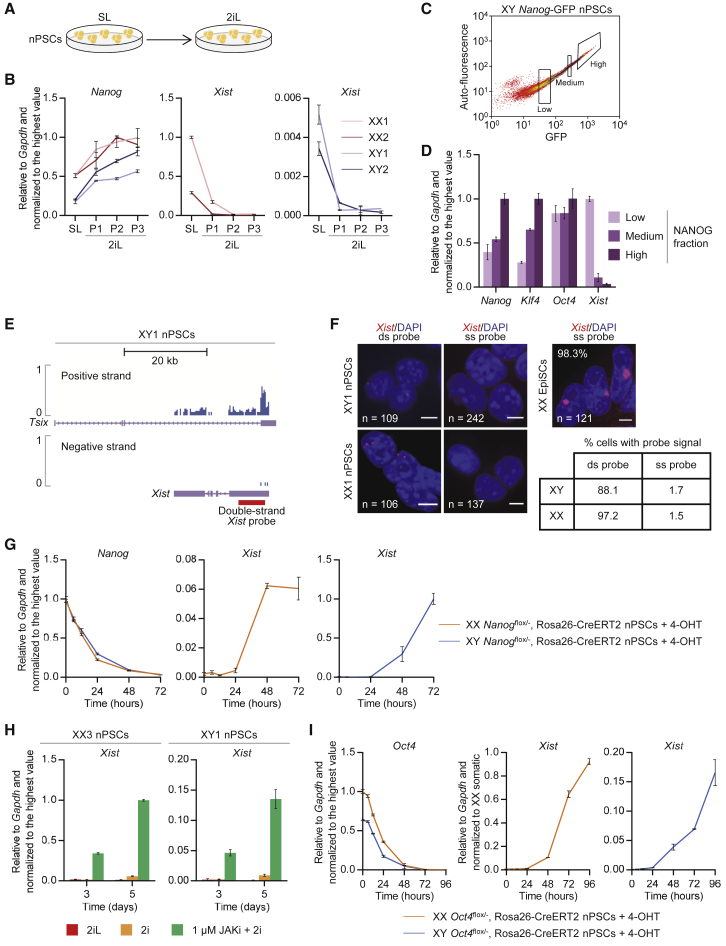
*Xist* Expression Is Abolished by a Robust Naive Pluripotent Network (A) Schematic illustrating the experiment performed to evaluate the impact of the nPSC culture conditions on the expression of *Xist*. (B) qRT-PCR analysis of *Nanog* and *Xist* in XX1, XX2, XY1, and XY2 ESC lines in SL versus 2iL. P indicates number of passages in 2iL. Error bars represent ± SD. (C) Flow cytometry analysis of male SL *Nanog*-GFP ESCs and subsequent sorting into three *Nanog*-GFP populations: low, medium, and high. (D) qRT-PCR analysis of *Nanog*, *Klf4*, *Oct4*, and *Xist* in low, medium, and high *Nanog*-GFP ESCs. Error bars represent ± SD. (E) Strand-specific RNA-seq showing expression of the positive and negative strands at the *Xist* locus in male 2iL ESCs. The double-strand *Xist* probe used in (F) is represented in red. (F) RNA FISH in male and female 2iL ESCs with a double-strand (ds) probe (left) or with a single-strand (ss) probe detecting only *Xist* (right). The percentage of cells with probe signal is indicated. Female EpiSCs were used as a control for the ss probe. The scale bar represents 5 μm. (G) qRT-PCR analysis of *Nanog* and *Xist* in female and male *Nanog*^flox/−^, Rosa26-CreERT2 ESCs in 2iL at indicated time points following treatment with 4-OHT. Error bars represent ± SD. (H) qRT-PCR analysis of *Xist* in XX3 and XY1 ESCs in 2iL, 2i or after 3 and 5 days in 1 μM JAKi + 2i. Error bars represent ± SD. (I) qRT-PCR analysis of *Oct4* and *Xist* in female and male *Oct4*^flox/−^, Rosa26-CreERT2 ESCs in 2iL at indicated time points following treatment with 4-OHT. Female somatic cells were used as control for *Xist* expression. Error bars represent ± SD.

To evaluate the impact of gene expression homogeneity and increased naive pluripotent gene expression on the levels of *Xist*, we analyzed two male and two female SL-derived embryonic stem cell (ESC) lines before and after passaging in 2iL (Figures 1A, S1A, and S1B). As expected, upregulation of naive pluripotent network components was observed after transferring cells from SL to 2iL (Figures 1B and S1C). Remarkably, 2iL conditions led to repression of *Xist* in both male and female ESCs after only one passage (Figure 1B).

ESCs in SL present heterogeneous levels of naive markers (Chambers et al., 2007), rendering these cells a useful tool to study the relationship between naive network status and *Xist* expression. Male SL ESCs with *Nanog*-GFP reporter were sorted into low, medium, and high GFP populations (Figures 1C and S1D). *Oct4* levels were maintained in all three fractions, whereas the pluripotency factor *Klf4* positively correlated with *Nanog* expression (Figure 1D). Interestingly, *Xist* expression was 28-fold lower in *Nanog*-high than *Nanog*-low cells (Figure 1D).

To validate the qRT-PCR data, we performed strand-specific RNA sequencing (RNA-seq) in 2iL-cultured male nPSCs. This clearly showed that expression at the *Xist* locus was exclusively antisense (Figure 1E). We also analyzed the pattern of *Xist* by RNA fluorescence *in situ* hybridization (FISH) using a single-strand (ss) *Xist*-specific probe and a conventional double-strand (ds) probe detecting *Xist* exon 1 and also any present antisense transcript. When using the ds probe and depending on the gender, one or two pinpoints were detected in 88% or 97% of cells, respectively (Figure 1F). In contrast, with the ss probe, *Xist* was detected in less than 2% of cells (Figure 1F). Together, these data demonstrate that *Xist* is not expressed in robust self-renewing nPSCs.

2iL culture medium allows the removal of otherwise essential components of the naive TF network (Ying et al., 2008). Thus, we assessed the effect of the withdrawal of these on *Xist* expression over 3 days. We performed 4-hydroxytamoxifen (4-OHT)-mediated Cre deletion of *Nanog* from *Nanog*^flox/−^, Rosa26-CreERT2 ESCs (Figures 1G and S1F–S1H). We found an inverse correlation between the expression of *Nanog* and *Xist*, which was highly upregulated in both females and males (Figure 1G). As expected, *Nanog* deletion also had an effect on the expression of its downstream targets such as *Klf4* and *Esrrb*, but not on *Oct4* or other naive pluripotency genes (Figure S1H).

JAK/STAT3 signaling plays an important role in the maintenance of pluripotency (Martello and Smith, 2014). In addition, activated STAT3 (p-STAT3) and its downstream target genes have binding sites within the X-inactivation center (XIC) that harbors *Xist* and other genes involved in its regulation (Sánchez-Castillo et al., 2015). Here, inhibition of JAK/STAT3 signaling was achieved by treatment of ESCs with a JAK inhibitor (JAKi) and confirmed by depletion of p-STAT3 (Figures 1H, S1I, and S1J) and downregulation of its target *Socs3* (Figure S1K). Interestingly, treatment with 1 μM JAKi induced upregulation of *Xist* in both female and male ESC lines (Figure 1H).

Overall, these data show that perturbing the naive pluripotent network leads to a sharp upregulation of *Xist* in both male and female ESCs. However, inhibition of JAK/STAT3 signaling also impacted expression of naive factors, such as *Oct4* (Figure S1K), which are known to be required for the integrity of the pluripotency network. This raised the need to investigate whether the observed sharp upregulation of *Xist* in males is indeed associated with a weaker but nonetheless naive identity or/and a consequence of naive cell differentiation. To investigate this, we analyzed *Xist* expression kinetics upon 4-OHT-mediated Cre deletion of *Oct4* in male and female *Oct4*^flox/−^, Rosa26-CreERT2 ESCs (Figures 1I, S1L, S1M, and S1N). In this context, the naive network collapses and cells lose the naive identity. Importantly, concomitantly with *Oct4* deletion, both female and male cells exhibited a striking increase in the expression of *Xist* (Figure 1I). In males, this reached 16% of *Xist* female somatic cell levels.

Together, these data link the loss of naive gene expression and identity to the upregulation of *Xist*.

### *Xist* Is Highly Expressed in a Transient and Rapid Fashion at the Onset of Male nPSC Differentiation

**Figure 2:**
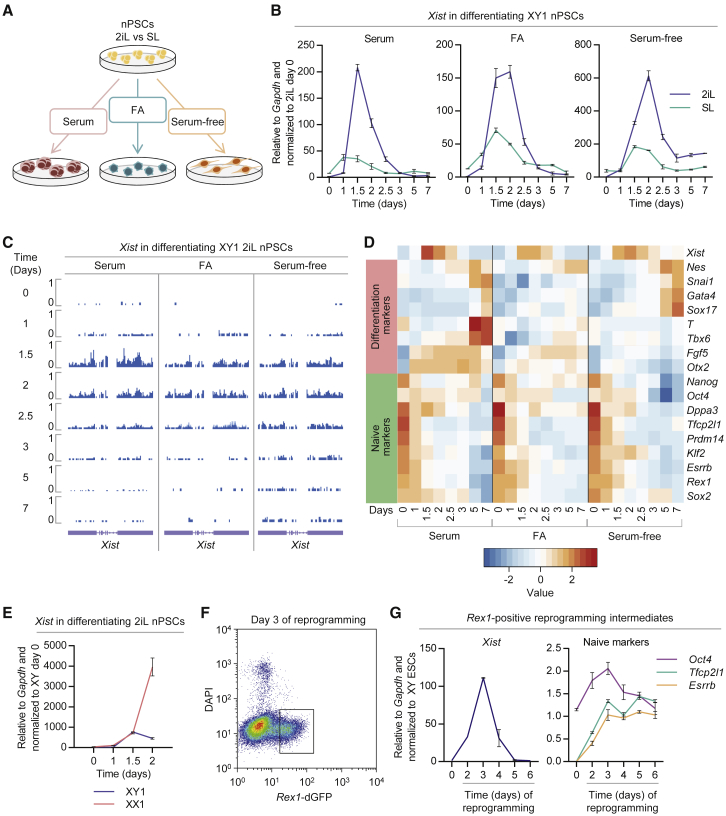
*Xist* Is Transiently and Rapidly Upregulated in Male nPSC Differentiation and Male EpiSC Reprogramming (A) Schematic illustrating three conditions employed to differentiate 2iL and SL nPSCs: suspension culture in serum to generate EBs or adherent monolayer culture in serum-free media ± Fgf2+ActivinA (FA). (B) qRT-PCR analysis of *Xist* during differentiation of male ESCs in three different conditions. Before differentiation, ESCs were maintained in 2iL or SL conditions, as indicated. Error bars represent ± SD. (C) Strand-specific RNA-seq (negative strand only) showing expression of *Xist* during differentiation of male 2iL ESCs in three different conditions. Scale represents reads per million (RPM). (D) Heatmap showing expression profile of *Xist*, differentiation markers, and naive markers during differentiation of male 2iL ESCs, as indicated. Scale represents *Z* scores of log2-transformed expression values. (E) qRT-PCR analysis of *Xist* during EB differentiation of male versus female 2iL ESCs. Error bars represent ± SD. (F) Flow cytometry analysis of male GY118F *Rex1*+/dGFP EpiSCs following reprogramming induction with GCSF in 2iL. Cells were sorted at different time points, with *Rex1*-dGFP reporter activation indicating the subset of cells successfully transitioning to the naive identity. A representative plot from day 3 is shown. (G) qRT-PCR analysis of *Xist* and naive markers (*Oct4*, *Tfcp2l1*, and *Esrrb*) in male *Rex1*-positive reprogramming intermediates at different time points after induction of reprogramming with 2iL+GCSF/GY118F. Parental EpiSCs (day 0) and ESCs in 2iL were used as controls. Error bars represent ± SD.

It is defined in the literature that *Xist* is repressed as male nPSCs undergo differentiation both *in vivo* and *in vitro* (Panning et al., 1997, Sheardown et al., 1997). Intriguingly, our results above showed a surprisingly high upregulation of *Xist* in males with a compromised naive network, leading us to revisit the kinetics of *Xist* expression during male nPSC differentiation. To address this, we induced differentiation of male ESCs that had previously been cultured in either traditional SL or optimal 2iL (Figure 2A). Surprisingly, a striking upregulation of *Xist* was observed in differentiating male 2iL ESCs (Figure 2B). Moreover, this was independent of the differentiation condition applied. *Xist* upregulation was transient and rapid, occurring between 1.5 and 2 days after induction of differentiation (Figure 2B). Although less apparent, this pattern was also observed in differentiating male SL ESCs (Figure 2B). These results were confirmed using independent male ESC and induced pluripotent stem cell (iPSC) lines (Figures S2A and S2B).

Recent reports highlighted that erosion of imprints can occur during the culture of nPSCs (Choi et al., 2017, Yagi et al., 2017). However, *Xist* is unlikely to have its expression pattern affected, as it is not an imprinted gene in either the naive epiblast or nPSCs.

Independent differentiation time courses were analyzed by strand-specific RNA-seq. In agreement with the qRT-PCR data, when male 2iL ESCs are differentiated, there is a surge of *Xist* expression that starts 1.5 days following induction of differentiation and lasts approximately 12 hr (Figure 2C). Principal-component analysis (PCA) based on differentially expressed genes showed that 2iL ESCs have a more undifferentiated starting point in relation to SL ESCs (Figure S2C), which corroborates our qRT-PCR data showing that transferring nPSCs from SL to 2iL increases the expression of naive pluripotency markers. The expression of known regulators of *Xist* was also analyzed. *Ftx*, *Jpx*, and *Tsx* also appear to be downregulated by 2iL conditions in comparison to SL at day 0 (Figure S2D). Upon differentiation, positive and negative regulators of *Xist* are also transiently upregulated (Figures S2D–S2F). Global analysis of the expression pattern of long non-coding RNAs showed that *Xist* follows a pattern during differentiation that is distinct from other long non-coding RNAs (Figure S3A).

To position *Xist* upregulation in the sequence of events taking place during differentiation, we compared its timing relative to naive pluripotent and lineage marker gene expression (Figure 2D). Importantly, downregulation of the naive pluripotency factors *Sox2*, *Nanog*, *Rex1*, *Esrrb*, *Klf2*, *Prdm14*, and *Tfcp2l1* preceded *Xist* upregulation. In contrast, expression of lineage markers was detected only after *Xist* upregulation. In combination with the effects of naive network perturbations on *Xist* expression (Figure 1), these results indicate that the upregulation of *Xist* observed in male cells upon induction of differentiation is caused by a weakening naive pluripotent network.

The differentiation experiments were also performed in a female ESC line. Remarkably, 1.5 days after induction of embryoid body (EB) differentiation, *Xist* expression reaches very similar levels in male and female cells (Figure 2E). However, while *Xist* expression keeps increasing in female cells, it starts declining in male cells.

Together, these results reveal that at the onset of differentiation, males upregulate *Xist* at a level similar to that of females.

### *Xist* Is Transiently Upregulated during Reprogramming of Male Cells

The aforementioned mechanistic association between a functional naive TF network and the repression of *Xist* led us to investigate whether these are correlated in the context of reprogramming to naive pluripotency in 2iL. We analyzed the productive reprogramming intermediates of male EpiSCs using STAT3 activation (Yang et al., 2010) and *Rex1*-dGFP reporter activity (Kalkan et al., 2017) to induce and monitor reprogramming, respectively (Figures 2F and S3B). As expected, *Xist* was repressed in both male EpiSCs and control ESCs (Figure 2G). However, in productive reprogramming intermediates, *Xist* was sharply upregulated (Figure 2G) prior to establishment of a consolidated nPSC identity. It is possible that this is due to loss of tight control of gene expression. Alternatively, it could reflect the fragile nature of the nascent naive network during reprogramming.

### Male X Chromosome Exhibits Hallmarks of XCI upon *Xist* Upregulation

**Figure 3:**
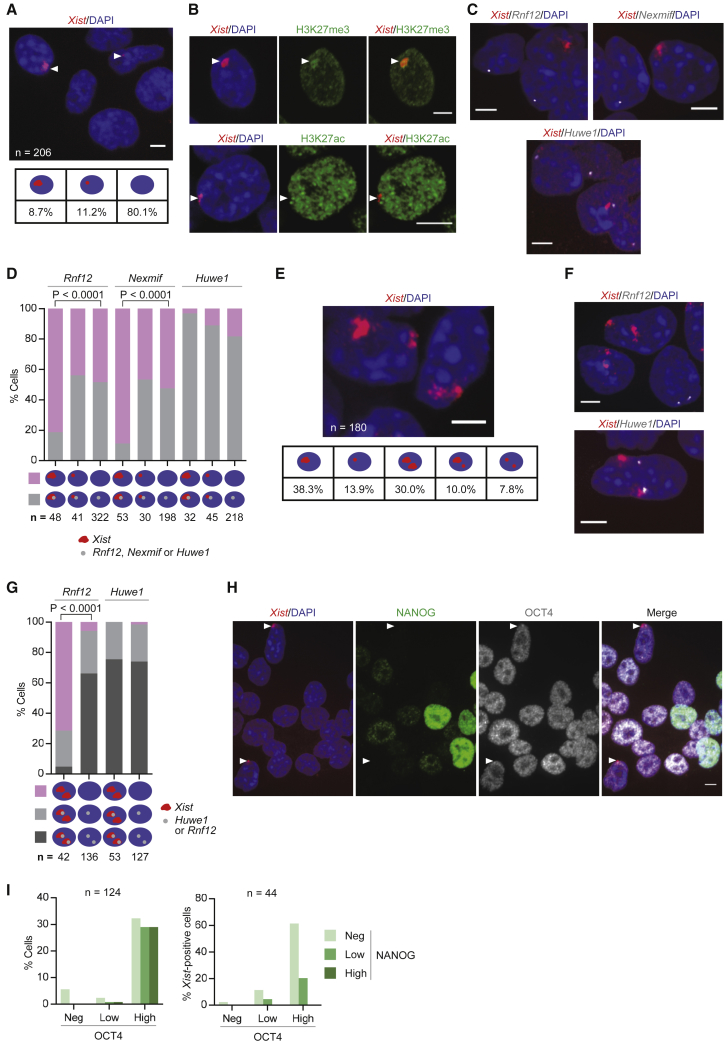
Males Undergo Transient XCI (A) RNA FISH for *Xist* (red) in male 2iL ESCs at 1.5 days of differentiation in FA using a strand-specific probe. White arrowheads indicate *Xist* signals. Quantification of the different *Xist* RNA patterns is shown. (B) Immuno-RNA FISH for *Xist* (red) and H3K27me3 or H3K27ac (green) in male 2iL ESCs at 1.5 days of differentiation in FA. White arrowheads indicate *Xist* cloud. (C) RNA FISH for *Xist* (red) and *Rnf12*, *Nexmif*, or *Huwe1* (grayscale) in male 2iL ESCs at 1.5 days of differentiation in FA. (D) Quantification of RNA FISH patterns for the X-linked genes *Rnf12*, *Nexmif*, or *Huwe1* and *Xist* as shown in (C). Gray indicates the presence of *Rnf12*/*Nexmif*/*Huwe1* signal, and pink indicates the absence of *Rnf12*/*Nexmif*/*Huwe1* signal. (E) RNA FISH for *Xist* (red) in female 2iL ESCs at 1.5 days of differentiation in FA using ss probe. Quantification of different *Xist* RNA patterns is shown. (F) RNA FISH for *Xist* (red) and *Rnf12* or *Huwe1* (grayscale) in female 2iL ESCs at 1.5 days of differentiation in FA. (G) Quantification of RNA FISH patterns for X-linked genes *Rnf12* or *Huwe1* and *Xist* as shown in (F). Dark gray indicates biallelic *Rnf12*/*Huwe1* signal, light gray indicates monoallelic *Rnf12*/*Huwe1* signal, and pink indicates the absence of *Rnf12*/*Huwe1* signal. (H) Immuno-RNA FISH for *Xist* (red), NANOG (green), and OCT4 (grayscale) in male 2iL ESCs at 1.5 days of differentiation in FA. White arrowheads indicate *Xist* clouds. (I) Percentage of NANOG- and OCT4-expressing cells in the population (left) and in cells exhibiting *Xist* cloud (right) as shown in (H). Fisher’s exact test was used for statistical analysis. ESC lines used were XY1 and XX1. Scale bar represents 5 μm.

Next, we analyzed whether the observed *Xist* upregulation in males was linked to events associated with the initiation of XCI as previously described for females. To look at this, we assessed the nuclear pattern of *Xist* by RNA FISH. Surprisingly, 1.5 days after male 2iL ESCs were induced to differentiate in FA, 20% of cells showed *Xist* RNA expression. Interestingly, nearly half of these exhibited *Xist* RNA clouds, with various sizes, characteristic of XCI (Figure 3A). Although in a smaller proportion, male SL ESCs induced to differentiate also exhibited cells showing *Xist* RNA accumulation (Figure S4A). Appearance of an *Xist* RNA cloud during male nPSC differentiation has been reported before (Monkhorst et al., 2008). However, the percentage of cells exhibiting this pattern was less than 0.5%, a much smaller proportion than reported in this study. The difference in the results could be explained by the homogeneity and robust self-renewal of our 2iL nPSC cultures and by the choice of time point to conduct the analysis.

Another event associated with XCI in females is accumulation of the repressive histone mark trimethyl-H3K27 (H3K27me3) (Plath et al., 2003, Silva et al., 2003) on the future Xi. Interestingly, H3K27me3 accumulation was observed in 63% of male cells showing *Xist* RNA cloud (Figures 3B, S4B, and S4C).

We then addressed the status of acetyl-H3K27 (H3K27ac), which is a mark of active enhancers (Creyghton et al., 2010). This was found hypoacetylated at the male *Xist* RNA cloud (Figures 3B, S4B, and S4D). Together, these results indicate that a silencing epigenetic signature has formed in the *Xist*-coated male X chromosome.

To examine whether *Xist* accumulation was inducing gene silencing, we performed RNA FISH analysis for nascent transcripts of 5 X-linked genes (*Rnf12*, *Nexmif*, *Nsdhl*, *Wbp5*, and *Huwe1*). The percentage of cells expressing *Rnf12*, *Nsdhl*, and *Nexmif* was between 3- and 4-fold lower in male cells showing *Xist* cloud that in male cells lacking *Xist* accumulation, and in the case of *Wbp5*, no examples of gene expression were found at the male X chromosome coated by *Xist* (Figures 3C, 3D, and S4E). In contrast, *Huwe1* showed no difference. *Rnf12*, *Nsdhl*, *Wbp5*, and *Nexmif* are in closer proximity to the *Xist* locus than *Huwe1* and are also known to be silenced early during XCI, unlike *Huwe1*, which is silenced at a late stage (Marks et al., 2015). At the cell population level, *Nexmif* was also downregulated (Figure S4F). However, *Xist*-mediated silencing did not affect population levels of *Rnf12* expression (Figure S4F). These data are consistent with a rapid transient *Xist* upregulation in males, a transience that is sufficient to have an impact on early-silencing genes but not lasting long enough to affect late-silencing ones.

Together these results show that males undergo XCI. which had so far only been associated with females. However, unlike females, male XCI is partial, transient, and rapid.

### Females Exhibit Partial XCI of Both X Chromosomes at the Onset of Differentiation

The observed upregulation of *Xist* in males, which possess only one X chromosome, questions the existence of a choice mechanism preceding initiation of XCI in females. To address this, we performed *Xist* RNA FISH in differentiating female nPSCs. Analysis of these cells in 2iL self-renewing conditions showed that *Xist* is not expressed (Figure 1F). Strikingly, at 1.5 days of differentiation, we observed that 30% of the female cells expressing *Xist* display RNA accumulation on both X chromosomes (Figure 3E). Control experiments revealed that 99% of the cells in this female line have no more than two X chromosomes, demonstrating that these results cannot be explained by the presence of tetraploid cells in culture (Figures S1B and S4G).

To assess whether *Xist* accumulation is inducing gene silencing on both X chromosomes, we performed RNA FISH analysis for nascent transcripts of *Rnf12* and *Huwe1* (Figures 3F and 3G). The incidence of biallelic silencing of *Rnf12* was 12-fold greater in female cells exhibiting *Xist* RNA accumulation on both X chromosomes than in cells not expressing *Xist*, while *Huwe1* showed no difference.

These results indicate that the choice of which X chromosome is going to be irreversibly silenced may follow rather than precede the initiation of XCI.

### Transient *Xist* RNA Patterns Occur as Differentiating Cells Become NANOG Negative

To investigate whether *Xist* upregulation is consistent with a particular differentiation stage, we analyzed the expression of NANOG and OCT4 relative to *Xist* at the single-cell level. Due to asynchrony in nPSC differentiation, NANOG expression was variable while most cells remained OCT4-high at 1.5 days. Interestingly, *Xist* upregulation in males occurred in cells that were NANOG-low or -negative (Figures 3H and 3I). Likewise, we found biallelic *Xist* upregulation in female cells that were OCT4-high/NANOG-low or -negative (Figures S4H and S4I). The expression of active CASPASE-3, which is an early marker of apoptosis, was evaluated to confirm the viability of the *Xist*-positive male cells. Importantly, all analyzed *Xist*-positive cells were negative for active CASPASE-3 (Figure S4J).

These results are in agreement with a transient *Xist* upregulation occurring in a defined developmental window and in close relationship with the downregulation of the naive network.

### *In Vivo* Transient *Xist* Expression in Males and in Females on Both X Chromosomes

**Figure 4:**
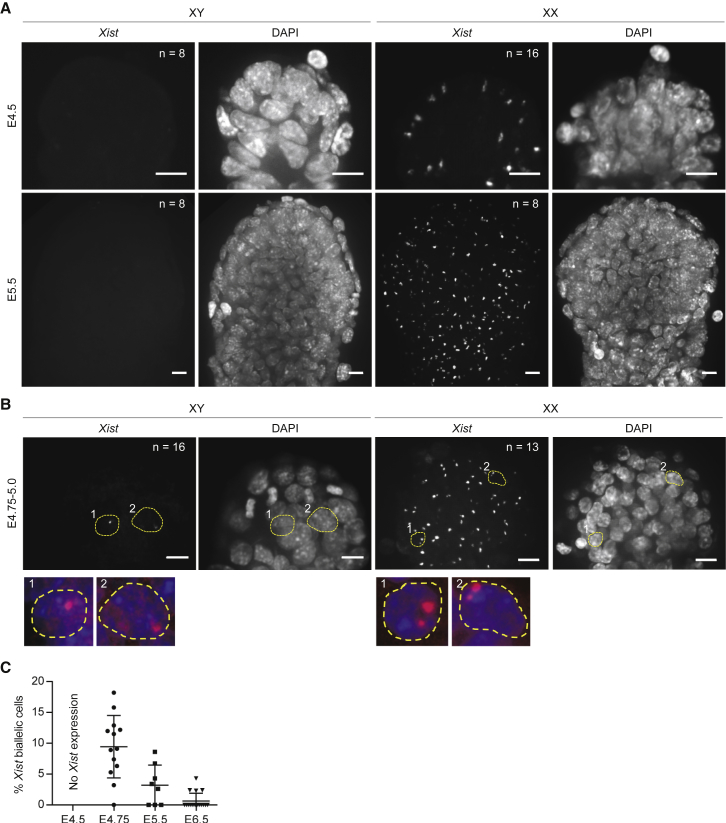
*Xist* Is Transiently Upregulated Monoallelically in Males and Biallelically in Females *In Vivo* (A and B) RNA FISH for *Xist* in representative male and female epiblasts of embryos at E4.5 and E5.5 (A) and at E4.75–E5.0 (B). (B) Examples of cells with monoallelic *Xist* expression in males and biallelic expression in females are delineated with yellow dashed lines. Higher magnification of these cells is displayed in the bottom panels. (C) Percentage of cells with biallelic *Xist* over total number of epiblast cells expressing *Xist* in female embryos at indicated developmental stages. Error bars represent ± SD. Scale bar represents 10 μm.

The embryonic day 4.5 (E4.5) naive epiblast is molecularly and functionally highly similar to *in vitro* 2iL nPSCs (Boroviak et al., 2015). Given the observed transient upregulation of *Xist* RNA during nPSC differentiation, we investigated whether this also occurs during *in vivo* differentiation of the pluripotent naive epiblast. In agreement with our findings in nPSCs, we found that E4.5 naive epiblast cells do not display *Xist* expression in either sex (Figure 4A). From E4.5, the embryo starts implanting and naive pluripotent gene expression is rapidly downregulated (Boroviak et al., 2015). Thus, we analyzed E5.5 male and female post-implantation embryos as a naive epiblast differentiation time point. At E5.5, we also failed to detect *Xist* expression in males, whereas female cells already exhibited one *Xist* RNA cloud in nearly all cells, which is the pattern associated with female differentiated cells. This led us to analyze earlier time points. Interestingly, at E4.75–E5.0, we found 1–3 cells per male epiblast exhibiting *Xist* RNA expression (Figure 4B). When analyzing female epiblast cells for the same time points, we observed that *Xist* was biallelically expressed in 10% of the epiblast cells showing *Xist* expression (Figures 4B and 4C). Together, these data are indicative that at implantation stage, and correlating with the downregulation of the naive epiblast network, male and female epiblast cells undergo transient and rapid monoallelic and biallelic expression of *Xist* respectively.

## Discussion

Our results show that *Xist* is fully repressed in both male and female nPSCs provided they have a robust naive TF network. This may be the result of a combination of direct and indirect mechanisms at the XIC, which contains multiple genomic binding sites occupied by naive pluripotent-associated TFs located at the *Xist* locus and at other non-coding and coding genes involved in *Xist* regulation (Sánchez-Castillo et al., 2015).

We showed that *Xist* accumulates transiently at the male X chromosome and induces partial epigenetic and transcriptional silencing in early differentiating cells. We have also demonstrated that this is linked to downregulation of the naive TF network. In this context, known positive regulators of *Xist*, such as *Jpx* (Tian et al., 2010), *Ftx* (Chureau et al., 2011), and RNF12 (Jonkers et al., 2009, Barakat et al., 2017), may transiently gain the upper hand and drive *Xist* expression. However, *Xist* is subsequently rapidly suppressed, suggesting that other mechanisms of *Xist* silencing are readily available. In agreement with this, deletion of *Tsix*, one of the non-coding RNAs implicated in *Xist* silencing, was previously found to correlate with the presence of an *Xist* RNA cloud in a proportion of differentiating male nPSCs (Sado et al., 2002). Likewise, *Dnmt1* mutant nPSCs exhibit *Xist* upregulation and silencing of X-linked genes in differentiating male cells (Beard et al., 1995, Panning and Jaenisch, 1996).

It has been proposed that initiation of XCI is related to the X chromosome/ploidy ratio (Monkhorst et al., 2008). Our study proposes downregulation of the naive TF network as the trigger for the initiation of XCI. It will now be interesting to investigate how these relate to each other.

XCI is defined as having five key phases in the following order: counting, choice, initiation, spreading, and maintenance (Augui et al., 2011). However, our data suggest that counting and choice between the two X chromosomes is not a requirement for XCI initiation. Furthermore, we show that the initiation and spreading phases also occur in males. Importantly, the initiation and spreading of XCI in males take place within a defined window of time where the process of XCI is known to be reversible (Wutz and Jaenisch, 2000), meaning that as long as *Xist* expression ceases within 3 days of differentiation, any induced changes on the X chromosome are fully reversible. Consistent with this, our data showed that *Xist* expression was already downregulated by day 3 of male cell differentiation. Therefore, we hypothesize that in the mouse species a counting mechanism, which relies on gender differences during the reversible period, occurs after XCI initiation.

It has been proposed that initiation of XCI in females is preceded by pairing of the two XIC loci and that the process of choice was dependent on pairing, therefore restricting XCI to female cells (Bacher et al., 2006, Xu et al., 2006). Moreover, although the negative regulation of *Xist* by the naive TF network is the same in male and female cells, the X-linked *Xist* activators adjacent to *Xist* and known to also work in *trans* will be duplicated in females. These and other mechanisms must somehow ensure that all but one X chromosome undergo irreversible XCI. It will now be important to further understand how these mechanisms allow XCI to be maintained in females only.

Human naive-like cells and human embryos cultured *in vitro* were found to have an intriguing *Xist* pattern (Petropoulos et al., 2016, Sahakyan et al., 2017, Vallot et al., 2017). In both cases, the presence of one or two *Xist* RNA clouds was reported for a proportion of male and female cells, respectively. As suggested by the authors, this may indicate species differences. However, it may also be akin to what we are reporting here, that is, as a result of a perturbed naive TF network, human naive cells may exhibit monoallelic and biallelic *Xist* upregulation in both males and females respectively.

In conclusion, our study redefines the paradigm of XCI and opens up new avenues to investigate how this process is regulated.

## STAR★Methods

### Key Resources Table

**Table:** 

REAGENT or RESOURCE	SOURCE	IDENTIFIER
**Antibodies**		

Monoclonal mouse anti-alpha-Tubulin	Abcam	Cat# ab7291, RRID:AB_2241126
Polyclonal rabbit anti-Nanog	Bethyl Laboratories	Cat# A300-397A, RRID:AB_386108
Monoclonal rat anti-Nanog	ThermoFisher Scientific	Cat# 14-5761-80, RRID:AB_763613
Monoclonal rabbit anti-Oct4	Cell Signaling Technology	Cat# 83932, RRID:AB_2721046
Polyclonal goat anti-Oct4	Santa Cruz Biotechnology	Cat# sc-8628, RRID:AB_653551
Monoclonal rabbit anti-Phospho-Stat3 (Tyr705)	Cell Signaling Technology	Cat# 9145, RRID:AB_2491009
Polyclonal rabbit anti-H3K27me3	Merck Millipore	Cat# 07-449, RRID:AB_310624
Polyclonal rabbit anti-H3K37ac	Abcam	Cat# ab4729, RRID:AB_2118291
Monoclonal rabbit anti-Cleaved Caspase-3 (Asp175)	Cell Signaling Technology	Cat# 9664, RRID:AB_2070042
Polyclonal rabbit anti-Rnf12	Merck Millipore	Cat# ABE1949, RRID:AB_2721047
HPR-conjugated donkey anti-rabbit	GE Healthcare	Cat# NA934, RRID:AB_772206
HPR-conjugated sheep anti-mouse	GE Healthcare	Cat# NA931, RRID:AB_772210
HPR-conjugated donkey anti-goat	Santa Cruz Biotechnology	Cat# sc-2020, RRID:AB_631728
Donkey anti-rabbit IgG (H+L) Highly Cross-Adsorbed Secondary Antibody, Alexa Fluor 488	ThermoFisher Scientific	Cat# A-21206, RRID:AB_2535792
Donkey anti-Rabbit IgG (H+L) Highly Cross-Adsorbed Secondary Antibody, Alexa Fluor 647	ThermoFisher Scientific	Cat# A-31573, RRID:AB_2536183
Donkey Anti-Rat IgG (H+L) Highly Cross-Adsorbed Secondary Antibody, Alexa Fluor 488	ThermoFisher Scientific	Cat# A-21208, RRID:AB_2535794
Goat biotinylated anti-Avidin	Vector Laboratories	Cat# BA-0300, RRID:AB_2336108

**Chemicals, Peptides, and Recombinant Proteins**		

N2	Cambridge Stem Cell Institute	N/A
B27	ThermoFisher Scientific	Cat# 17504044
Murine LIF	Hyvönen lab, Cambridge	N/A
CHIR99021	Stewart lab, Dresden	N/A
PD0325901	Stewart lab, Dresden	N/A
FBS	Labtech	Cat# FB-1001S/500
Egf	Peprotech	Cat# 315-09
Fgf2	Hyvönen lab, Cambridge	N/A
Activin A	Hyvönen lab, Cambridge	N/A
XAV 939	Tocris, Bio-techne	Cat# 3748
4-Hydroxytamoxifen	Sigma-Aldrich	Cat# 7904
InSolution JAK Inhibitor I	Merck-Millipore	Cat# 420097
GCSF	Peprotech	Cat# 300-23
KaryoMAX Colcemid Solution in HBSS	ThermoFisher Scientific	*Cat# 15210040*
*Taq* DNA Polymerase	QIAGEN	*Cat# 201205*

**Critical Commercial Assays**		

DNeasy Blood & Tissue Kit	QIAGEN	Cat# 69504
RNeasy Mini Kit	QIAGEN	Cat# 74104
SuperScript III First-Strand Synthesis SuperMix	ThermoFisher Scientific	Cat# 18080400
TaqMan Fast Universal PCR Master Mix (2X), no AmpErase UNG	ThermoFisher Scientific	Cat# 4352042
Esrrb TaqMan Gene Expression Assay	ThermoFisher Scientific	Mm00442411_m1
Ftx TaqMan Gene Expression Assay	ThermoFisher Scientific	Mm03455830_m1
Gapdh TaqMan Gene Expression Assay	ThermoFisher Scientific	4352339E
Klf2 TaqMan Gene Expression Assay	ThermoFisher Scientific	Mm01244979_g1
Klf4 TaqMan Gene Expression Assay	ThermoFisher Scientific	Mm00516104_m1
Klf5 TaqMan Gene Expression Assay	ThermoFisher Scientific	Mm00456521_m1
Nanog TaqMan Gene Expression Assay	ThermoFisher Scientific	Mm02384862_g1
Nexmif TaqMan Gene Expression Assay	ThermoFisher Scientific	Mm01239465_g1
Oct4 TaqMan Gene Expression Assay	ThermoFisher Scientific	Mm00658129_gH
Rex1 TaqMan Gene Expression Assay	ThermoFisher Scientific	Mm03053975_g1
Rnf12 TaqMan Gene Expression Assay	ThermoFisher Scientific	Mm00488044_m1
Socs3 TaqMan Gene Expression Assay	ThermoFisher Scientific	Mm01249143_g1
Sox2 TaqMan Gene Expression Assay	ThermoFisher Scientific	Mm03053810_s1
Tfcp2l1 TaqMan Gene Expression Assay	ThermoFisher Scientific	Mm00470119_m1
Xist TaqMan Gene Expression Assay	ThermoFisher Scientific	Mm01232884_m1
Biotin-Nick Translation Mix	Sigma-Aldrich	Cat# 11745824910
Illustra MicroSpin S-300 HR columns	GE Healthcare	Cat# 27513001
Salmon Sperm DNA, sheared	ThermoFisher Scientific	*Cat# AM9680*
Mouse Cot-1 DNA	ThermoFisher Scientific	*18440016*
Ribonucleoside Vanadyl Complex	New England Biolabs	*S1402S*
Texas Red Avidin DCS	Vector Laboratories	*A-2016*
Mouse Xist Stellaris RNA FISH Probe with Quasar 570 Dye	BioSearch Technologies	Cat# SMF-3011-1
Mouse Xist Stellaris RNA FISH Probe with Quasar 670 Dye	BioSearch Technologies	Cat# VSMF-3095-5
Custom Stellaris RNA FISH Probe with FISH Probe with Quasar 570 Dye	BioSearch Technologies	Cat# SMF-1063-5
Mouse Chromosome X Whole Chromosome Painting Probe, Green Label	MetaSystems Probes	Cat# D-1420-050-FI
Mouse Chromosome Y Whole Chromosome Painting Probe, Orange Label	MetaSystems Probes	Cat# D-1421-050-OR

**Deposited Data**		

RNA seq data	This paper	GEO: GSE109173

**Experimental Models: Cell Lines**		

E14tg2a ESC line	Smith lab, Cambridge	N/A
EFC ESC line	Smith lab, Cambridge	N/A
LF1 ESC line	Smith lab, Cambridge	N/A
LF2 ESC line	Smith lab, Cambridge	N/A
C6 ESC line	Smith lab, Cambridge	N/A
Nanog^flox/−^, Rosa26-CreERT2 ESC line	This paper	N/A
Oct4^flox/−^, Rosa26-CreERT2 ESC line	This paper	N/A
Rex1-dGFP EpiSC line	This paper	N/A
Rex1-dGFP NSC line	This paper	N/A
Nanog-GFP EpiSC line	Smith lab, Cambridge	N/A

**Experimental Models: Organisms/Strains**		

Mouse CD-1	Charles River	Cat# 022

**Oligonucleotides**		

Gender PCR primer Ube1XA (5′ to 3′): TGGTC TGGACCCAAACGCTGTCCACA	(Chuma and Nakatsuji, 2001)	N/A
Gender PCR primer Ube1XB (5′ to 3′): GGCA GCAGCCATCACATAATCCAGATG	(Chuma and Nakatsuji, 2001)	N/A

**Software and Algorithms**		

Fiji	Open Source	http://imagej.net/Fiji/Downloads
R	The R Project	https://www.r-project.org/
GraphPad Prism 6	GraphPad Software	https://www.graphpad.com/scientific-software/prism/
FlowJo	FlowJo, LLC	https://www.flowjo.com/
TrimGalore	Babraham Institute	http://www.bioinformatics.babraham.ac.uk/projects/trim_galore
TopHat2	Johns Hopkins University	https://ccb.jhu.edu/software/tophat
featureCounts	Walter and Eliza Hall Institute of Medical Research	http://bioinf.wehi.edu.au/featureCounts
DESeq2	Bioconductor	https://bioconductor.org/packages/release/bioc/html/DESeq2.html

### Contact for Reagent and Resource Sharing

Further information and requests for resources and reagents should be directed to and will be fulfilled by the Lead Contact, José Silva (jcs64@cam.ac.uk).

### Experimental Model and Subject Details

#### Cell lines

Male wild-type ESC lines included E14tg2a (XY1) and EFC (XY2). Female wild-type ESC lines included LF1 (XX1), LF2 (XX2) and C6 (XX3). The cell sorting experiment was performed in male *Nanog*-GFP ESCs. Female *Nanog*-GFP EpiSCs were used as control in *Xist* RNA FISH. *Nanog* deletion was performed in male and female *Nanog*^flox/-^, Rosa26-CreERT2 ESC lines. *Oct4* deletion was performed in male and female *Oct4*^flox/-^, Rosa26-CreERT2 ESC lines. Male iPSC line used was derived from *Rex1*-dGFP NSCs. Reprogramming was performed in male *Rex1*-dGFP EpiSCs.

#### Cell culture

Mouse ESCs and iPSCs were cultured in 2i+LIF (2iL), 2i, or serum+LIF (SL) as indicated. 2iL medium was composed of N2B27, 3 μM CHIR99021, 1 μM PD0325901 (Stewart lab, Dresden), and 20 ng ml^−1^ of murine LIF (Hyvönen lab, Cambridge). N2B27 medium comprised 1:1 DMEM/F-12 and Neurobasal (ThermoFisher Scientific), 2 mM L-glutamine (ThermoFisher Scientific), 1x penicillin-streptomycin (Sigma-Aldrich), 0.1 mM 2-mercaptoethanol (ThermoFisher Scientific), 1% B27 (ThermoFisher Scientific) and 0.5% N2 (homemade). SL medium contained GMEM (Sigma-Aldrich), 10% fetal bovine serum (Labtech), 1x non-essential amino acids (ThermoFisher Scientific), 1 mM sodium pyruvate (Sigma-Aldrich), 2 mM L-glutamine, 1X penicillin-streptomycin, 0.1 mM 2-mercaptoethanol and 20 ng ml^−1^ of LIF. EpiSCs were cultured in FAX medium composed of N2B27 supplemented with 12.5 ng ml^−1^ Fgf2, 20 ng ml^−1^ Activin A (Hyvönen lab, Cambridge) and 6.25 μg ml^−1^ XAV 939 (Bio-Techne). 4-OHT (Sigma-Aldrich) was used at a concentration of 500 nM and InSolution JAKi I (Merck Millipore) at a concentration of 1 μM. During expansion of *Nanog*^flox/-^ cell lines, selection with 200 μg ml^−1^ G418 (ThermoFisher Scientific) was applied to select for pluripotent cells based on *Nanog* promoter activity at the null allele. For ESCs and iPSCs, tissue-culture flasks were coated with 0.1% gelatin (Sigma-Aldrich) in PBS (Sigma-Aldrich). For EpiSCs, tissue-culture flasks were coated with 10 μg ml^−1^ fibronectin (Merck Millipore) in PBS (Sigma-Aldrich).

#### Cell differentiation

For embryoid body differentiation, 1.5 × 10^6^ cells were plated on 10 cm low-attachment dishes in serum-containing medium without LIF. For differentiation in adherent monolayer culture, 6 × 10^5^ cells were plated on gelatin-coated 10 cm dishes in serum-free (N2B27) or N2B27 supplemented with Fgf2 and Activin A (FA). XY1 and XX1 ESCs were not passaged more than 3 times in 2iL prior to the differentiation assays.

#### Reprogramming

Embryo-derived male EpiSCs with *Rex1*+/dGFP reporter (Kalkan et al., 2017) were used that constitutively express the GY118F receptor transgene known to drive EpiSC reprogramming via STAT3 activation (Yang et al., 2010). For reprogramming, EpiSCs were plated at 10,000 cells per fibronectin-coated 6-well in FAX maintenance medium. The following day, reprogramming was induced by switch to 2iL plus GCSF (30 ng ml^−1^ human GCSF, Peprotech). On days 2, 3, 4, 5 and 6, multiple reprogramming wells were harvested using accutase, stained with DAPI to eliminate nonviable cells, and sorted by flow cytometry to isolate the *Rex1*-dGFP-positive subpopulation for further analysis. Parental EpiSCs and male *Rex1*+/dGFP ESCs were used as negative and positive gating controls respectively.

#### Embryos

Embryos were collected from CD1 female mice (Charles River Laboratories, UK). Use of animals in this project was approved by the Animal Welfare and Ethical Review Body for the University of Cambridge (Procedure Project Licenses P76777883 and 80/2597).

### Method Details

#### RNA isolation, cDNA synthesis and qRT-PCR

Total RNA was isolated from cells using the RNeasy mini kit (QIAGEN) in accordance with the manufacturer’s protocol. One microgram of total RNA was reverse-transcribed using SuperScript III First-Strand Synthesis SuperMix (ThermoFisher Scientific). Reverse Transcription Quantitative Real-Time PCR (qRT-PCR) reactions were set up in triplicate using TaqMan Universal PCR Master Mix (ThermoFisher Scientific) and TaqMan gene expression assays (ThermoFisher Scientific). qRT-PCR experiments were performed on a StepOnePlus Real Time PCR System (ThermoFisher Scientific). Delta Ct (ΔCt) values compared to Gapdh were calculated and relative quantities calculated as 2 to the power of -ΔCt. The means of three values were calculated and normalized as indicated.

#### Flow cytometry

*Nanog*-GFP ESCs were resuspended in PBS containing 3.5% BSA (ThermoFisher Scientific) and sorting was performed using a MoFlo high-speed cell sorter (Beckman Coulter). A 514/10BP filter was used for GFP and a 580/30BP filter was used for autofluorescence. *Rex1*-dGFP positive reprogramming intermediates were sorted using a BD Influx 5 cell sorter (BD Biosciences). A 460/50 filter was used for DAPI and a 530/40 filter was used for GFP.

#### DNA extraction

In the case of the cell lines, DNA was isolated using the DNeasy Blood & Tissue Kit (QIAGEN) in accordance with the manufacturer’s protocol. In the case of the embryos, these were collected from the slides after RNA FISH and imaging and then lysed in 0.2% Triton X-100 and Proteinase K at 56°C for 10 minutes followed by 95°C for 15 minutes.

#### Gender PCR

Cell lines were sexed by PCR using primers Ube1XA and Ube1XB, which results in two products of distinct sizes from the Ube1x and Ube1y genes on the X and Y chromosome respectively (Chuma and Nakatsuji, 2001). PCR reactions were performed in a final volume of 20 μL with 100 ng of DNA, 1X CoralLoad buffer, 0.2 mM dNTPs, 0.35 μM primers and 2.5 units Taq DNA Polymerase (QIAGEN) and run on a thermal cycler with the following conditions: 94°C for 3 minutes, 30 cycles with 94°C for 30 s, 66°C for 30 s, and 72°C for 30 s, followed by 72°C for 10 minutes. In the case of the embryos 4 μL per embryo lysate were added to the PCR reaction. Products were electrophoresed on 2% agarose gel.

#### Western blotting

Dissociated cells were lysed in RIPA buffer (as described by Sigma-Aldrich) containing Complete-ULTRA protease-inhibitor and PhosStop phosphatase-inhibitor cocktails (Roche), and sonicated with Bioruptor200 (Diagenode) at high frequency, alternating 30 s on/off for 3 minutes. SDS-PAGE electrophoresis was performed using Bolt 10% Bis-Tris Plus gels (ThermoFisher Scientific) in a Novex MiniCell (ThermoFisher Scientific). Protein transfer was performed using semi-dry iBlot 2 system (ThermoFisher Scientific) and iBlot Transfer Stacks (ThermoFisher Scientific). The following primary antibodies dilutions were used: mouse monoclonal against α-Tubulin (1:5,000) from Abcam, rabbit polyclonal against NANOG (1:5,000) from Bethyl Laboratories, rabbit monoclonal against p-Y705-STAT3 (1:1,000) from Cell Signaling Technology, rabbit polyclonal against RNF12 (1:1,000) from Merck Millipore, and goat polyclonal against OCT4 (1:1,000) from Santa Cruz Biotechnology. Detection was achieved using HRP-linked secondary antibodies against the appropriate species (GE Healthcare) and ECL Plus Western Blotting Detection System (GE Healthcare).

#### *Xist* RNA FISH with double-stranded probe

Cells were plated on SuperFrost Plus Adhesion slides (ThermoFisher Scientific) and permeabilised in cytoskeletal buffer (100 mM NaCl, 300 mM sucrose, 3 mM MgCl2, 10 mM PIPES) containing 0.5% Triton X-100 (Sigma-Aldrich), 1 mM EGTA pH 8 and vanadyl ribonucleoside (New England Biolabs) for 5 minutes on ice. They were subsequently fixed in 4% paraformaldehyde (Sigma-Aldrich) for 10 minutes, briefly washed in 1X PBS and dehydrated through 70, 80, 95, and 100% ethanol, after which the slides were air-dried. At this stage, a denatured *Xist* probe was applied onto the slides and these were incubated overnight at 37°C.

The probe was prepared by labeling plasmid DNA containing a mouse *Xist* exon 1 fragment sequence (kindly provided by Professor Neil Brockdorff, University of Oxford, UK) with a Biotin-Nick Translation Mix (Sigma-Aldrich) according to manufacturer’s instructions and non-incorporated nucleotides were removed with Illustra MicroSpin S-300 HR columns (GE Healthcare). To 20 ng of probe, 10 μg of sheared salmon sperm DNA (ThermoFisher Scientific) and 3 μg of mouse Cot-1 DNA (ThermoFisher Scientific) were added. Finally, the probe was dehydrated by vacuum and resuspended in deionized formamide (VWR). Before applying to the slide, the probe was denatured at 80°C and 2X hybridization buffer (4X SSC, 20% dextran sulfate, 2 mg ml^-1^ BSA, 2 mM vanadyl ribonucleoside) was added.

The following day, the slides were washed at 42°C in 2X SSC/50% formamide for 15 minutes, then three times in 2X SSC for 5 minutes each. They were then transferred to 4X SSC/0.1% Tween 20 at room temperature and blocked in 4 mg ml^-1^ BSA in 4X SSC/0.1% Tween 20 at 37°C for 30 minutes. Probe detection was performed by first applying Avidin conjugated to Texas Red (1:500, Vector Laboratories), then a biotinylated anti-avidin antibody (1:200, Vector Laboratories), followed by a second layer of Avidin-Texas Red. All detection reagents were diluted in 4 mg ml^-1^ BSA in 4X SSC/0.1%Tween 20 and incubated at 37°C for 30 minutes followed by three washes in 4X SSC/0.1% Tween 20 in between each step. Finally, the slides were mounted in Vectashield Mounting Medium containing DAPI (Vector Laboratories).

#### RNA FISH with single-stranded probe

RNA FISH protocol was modified from the Stellaris (Biosearch Technologies) protocol for adherent mammalian cells. Cells were plated on SuperFrost Plus Adhesion slides (ThermoFisher Scientific) and fixed in 4% PFA (Sigma-Aldrich) at room temperature for 10 minutes. They were subsequently washed in 1X PBS and permeabilised in cytoskeletal buffer (100 mM NaCl, 300 mM sucrose, 3 mM MgCl2, 10 mM PIPES) containing 0.5% Triton X-100, 1 mM EGTA pH 8 and vanadyl ribonucleoside (New England Biolabs) for 5 minutes on ice. Following washing in 1X PBS, they were incubated in 70% ethanol at 4°C overnight. Cells were incubated in 10% formamide (VWR) in 2X SSC (Sigma-Aldrich) for 10 minutes and then in 250 nM Stellaris Probes diluted in 100 mg mL^−1^ dextran sulfate (MP Biomedicals) and 10% formamide in 2X SSC at 37°C overnight. *Xist* was recognized using Stellaris FISH Probes labeled with either Quasar 570 or Quasar 670 (BioSearch Technologies). *Rnf12*, *Nexmif*, *Nsdhl*, *Wbp5* and *Huwe1* were recognized using Custom Stellaris FISH Probes labeled with Quasar 570 (BioSearch Technologies). The sequences of 48 oligonucleotides designed against unique intronic sequences using the online Stellaris Probe Designer tool (BioSearch Technologies). The *Rnf12* and *Huwe1* probes sequences were kindly provided by Prof. Neil Brockdorff and Dr. Tatyana Nesterova (University of Oxford, UK). After hybridization, cells were incubated in 10% formamide in 2X SSC at 37°C for 30 minutes followed by a wash in 2X SSC at room temperature for 5 minutes. Cells were mounted with Vectashield Antifade Mounting Medium with DAPI (Vector Laboratories).

#### RNA FISH of mouse embryos

Mouse embryos were collected from CD1 mice and fixed in 4% PFA at room temperature for 15 minutes. Embryos were further processed for RNA FISH using Stellaris probes (BioSearch Technologies) according to the procedure described above.

#### Immunofluorescence

Immunofluorescence was always performed in combination with RNA FISH using Stellaris probes. Sequential immunofluorescence and RNA FISH protocol was modified from the Stellaris (Biosearch Technologies) protocol for adherent mammalian cells. Cells were fixed and permeabilised as for RNA FISH. They were then washed in 1X PBS and incubated with primary antibody diluted in 1X PBS at 37°C for 2 hours. Primary antibodies were used as follows: rabbit polyclonal against H3K27me3 (1:500) from Merck Millipore, rabbit polyclonal against H3K27ac (1:500) from Abcam, rat monoclonal against NANOG (1:300) from ThermoFisher Scientific, rabbit monoclonal against OCT4 (1:300) from Cell Signaling Technology and rabbit monoclonal against cleaved CASPASE-3 (1:100) from Cell Signaling Technology. Following washing in 1X PBS, cells were incubated with secondary antibody diluted in 1X PBS at 37°C for 1 hour. An appropriate Alexa Fluor conjugated secondary antibody (1:1,000) from ThermoFisher Scientific was used. Cells were then washed in 1X PBS and fixed again in 4% PFA at room temperature for 10 minutes. Following washing with 1X PBS, RNA FISH protocol was carried out as described above, starting from the incubation in 10% formamide in 2X SSC.

#### Metaphase spread

ESCs were plated onto a gelatinised 6-well 2 days prior preparation of the chromosome spreads. Cultures were then arrested in metaphase by addition of 0.5 μg mL^−1^ KaryoMAX colcemid (ThermoFisher Scientific) and incubation at 37°C for 3 hours. Cell were then washed in PBS, harvested with accutase and centrifuged at 300 g for 5 minutes. Following aspiration of the supernatant, the pellet was resuspended in 5 mL of 0.075 M KCl solution and incubated at 37°C for 15 minutes. Then 100 μL of ice cold methanol:glacial acetic acid (3:1) fixative solution were added drop-wise followed by an incubation on ice for 10 minutes. Following incubation, cells were centrifuged at 300 g for 5 minutes, supernatant was aspirated leaving 500 μL in the tube and 5 mL of fixative solution were added to the cells. Again 500 μL of the supernatant were kept in the tube after centrifugation and spread onto a glass slide. After at least 3 hours at room temperature, the slide was stained or stored at −20°C for further analysis.

#### DNA FISH chromosome painting

Prior to X and Y chromosome painting, immune RNA FISH was performed, slides were imaged and x-y coordinates were marked for future reference. After removal of the coverslip, slides were washed in 2X SSC at room temperature. X and Y chromosome painting was also performed on metaphase spreads. Slides were dehydrated through an ice-cold ethanol series (70%, 80%. 95% and 100%) for 3 minutes each and then allowed to air dry. X and/or Y chromosome paint probe (Metasystems) was added to the slide, denaturation was carried out at 75°C for 2 minutes and slides were incubated at 37°C overnight. After hybridization, slides were incubated in 0.4X SSC at 72°C for 2 minutes followed by a wash with 0.05% Tween-20 in 2X SSC at room temperature for 30 s. Slides were rinsed in water, allowed to air dry and mounted with Vectashield Antifade Mounting Medium with DAPI (Vector Laboratories).

#### Microscopy and image analysis

Images were taken with an Eclipse Ti Spinning Disk confocal microscope (Nikon) equipped with an Andor Revolution XD System using either 40X or 60X objectives. Images were processed and analyzed with ImageJ. Presented images are maximum intensity projections of Z stack slices or, in the case of embryos, selected Z stack slices. Scoring of RNA FISH signals was done by eye from images.

#### RNA-seq

RNA integrity was assessed on a Qubit Fluorometer (ThermoFisher Scientific) and Agilent Bioanalyzer Nano Chips (Agilent Technologies). Depletion of ribosomal RNA was performed on 2-5 μg of total RNA using the Ribo-Zero rRNA Removal Kit (Illumina) and libraries were produced from 10-100ng of ribosomal-depleted RNA using NextFlex Rapid Directional RNA-seq Kit (Bioo Scientific) with 12 cycles of PCR amplification. Libraries were pooled in equimolar quantities and sequenced on the HiSeq4000 platform (Illumina) at CRUK.

### Quantification and Statistical Analyses

Where indicated, statistical analysis was performed by Fisher’s exact test using GraphPad Prism. “n” values in figures represent the number of embryos or number of cells analyzed. All qRT-PCR data represent the mean of three technical replicates. All error bars represent ± standard deviation (SD).

#### RNA-seq analysis

RNA-seq reads were adaptor-trimmed with TrimGalore (http://www.bioinformatics.babraham.ac.uk/projects/trim_galore) and mapped to the mouse reference genome (GRCm38/mm10) with TopHat2 (https://ccb.jhu.edu/software/tophat) allowing for one mismatch and alignments guided by Ensembl gene models (Ensembl release 82). Strand-specific read counts were obtained with featureCounts (http://bioinf.wehi.edu.au/featureCounts). Transcript counts were normalized, and the statistical significance of differential expression between samples was assessed using the R Bioconductor DESeq2 package (https://bioconductor.org/packages/release/bioc/html/DESeq2.html). Transcript counts normalized by DESeq2 size factors were subsequently normalized by their length/1000. Principal component analysis (PCA) was performed by singular value composition using the R prcomp() function on scaled expression values.

### Data and Software Availability

The accession number for the RNA-seq data reported in this paper is GEO: GSE109173.

## Supplemental Information

Document S1. Figures S1–S4

Document S2. Article plus Supplemental Information
