# Supplementary material for: Exit from Naive Pluripotency Induces a Transient X Chromosome Inactivation-like State in Males
Source: Cell Stem Cell. 2018 Jun 1;22(6):919–928.e6. doi: 10.1016/j.stem.2018.05.001 (PMC5989057; doi:10.1016/j.stem.2018.05.001)
Supplement: Document S1. Figures S1–S4 [file mmc1.pdf]

Cell Stem Cell, Volume *22*

Supplemental Information

Exit from Naive Pluripotency Induces a Transient X Chromosome Inactivation-like State in Males

Elsa J. Sousa, Hannah T. Stuart, Lawrence E. Bates, Mohammadmersad Ghorbani, Jennifer Nichols, Sabine Dietmann, and José C.R. Silva

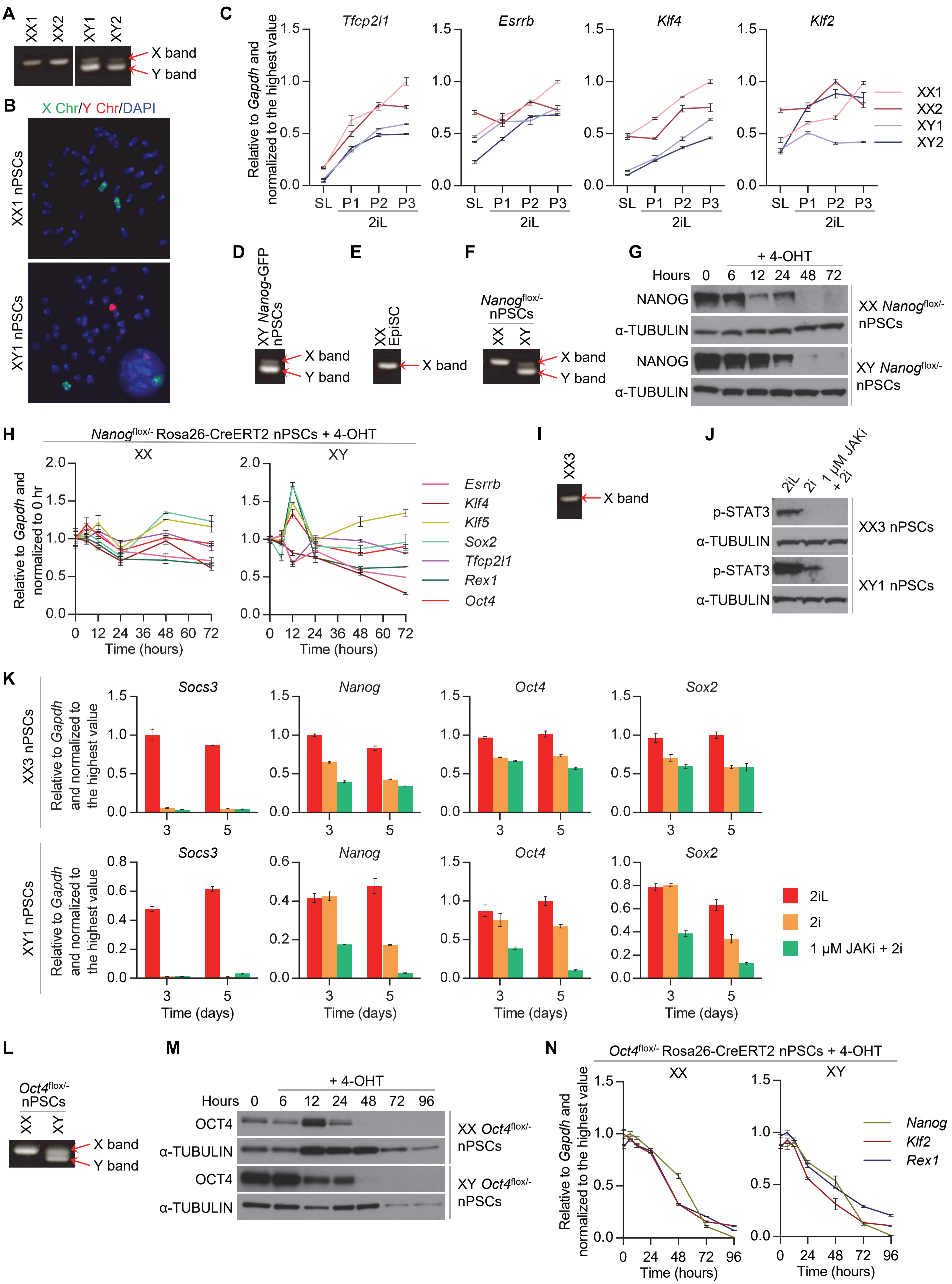

A
XX1 XX2 XY1 XY2
X band
Y band
B
X Chr/Y Chr/DAPI
XX1 nPSCs
XY1 nPSCs
C
Relative to Gapdh and normalized to the highest value
Tfcp2l1
Esrrb
Klf4
Klf2
SL P1 P2 P3
2iL
XX1
XX2
XY1
XY2
D
XY Nanog-GFP nPSCs
X band
Y band
E
XX EpiSC
X band
F
Nanogflox/- nPSCs
XX XY
X band
Y band
G
+ 4-OHT
Hours 0 6 12 24 48 72
NANOG
α-TUBULIN
XX Nanogflox/- nPSCs
XY Nanogflox/- nPSCs
H
Nanogflox/- Rosa26-CreERT2 nPSCs + 4-OHT
XX
XY
Relative to Gapdh and normalized to 0 hr
Time (hours)
Esrrb
Klf4
Klf5
Sox2
Tfcp2l1
Rex1
Oct4
I
XX3
X band
J
2iL 2i 1 μM JAKi + 2i
p-STAT3
α-TUBULIN
XX3 nPSCs
XY1 nPSCs
K
XX3 nPSCs
XY1 nPSCs
Relative to Gapdh and normalized to the highest value
Socs3
Nanog
Oct4
Sox2
Time (days)
2iL
2i
1 μM JAKi + 2i
L
Oct4flox/- nPSCs
XX XY
X band
Y band
M
+ 4-OHT
Hours 0 6 12 24 48 72 96
OCT4
α-TUBULIN
XX Oct4flox/- nPSCs
XY Oct4flox/- nPSCs
N
Oct4flox/- Rosa26-CreERT2 nPSCs + 4-OHT
XX
XY
Relative to Gapdh and normalized to the highest value
Time (hours)
Nanog
Klf2
Rex1

Figure S1. *Xist* expression is abolished by a robust naive pluripotent network, Related to Figure 1.

(A) PCR-based gender determination of the cell lines LF1 (XX1), LF2 (XX2), E14tg2a (XY1), and EFC (XY2).

(B) X (green) and Y (red) DNA FISH chromosome painting of metaphase spreads of the ESC lines LF1 (XX1) and E14tg2a (XY1).

(C) qRT-PCR analysis of naive markers (*Tfcp2l1*, *Esrrb*, *Klf4*, and *Klf2*) in two female, LF1 (XX1) and LF2 (XX2), and two male, E14tg2a (XY1) and EFC (XY2), ESC lines in SL versus 2iL conditions. P indicates the number of passages in 2iL. Data shown are the mean of 3 technical replicates. Error bars represent ± SD.

(D) PCR-based gender determination of the *Nanog*-GFP ESC line.

(E) PCR-based gender determination of the control EpiSC line.

(F) PCR-based gender determination of the *Nanog*$^{flox/-}$, Rosa26-CreERT2 ESC lines.

(G) Western blot analysis of NANOG in female and male *Nanog*$^{flox/-}$, Rosa26-CreERT2 ESCs in 2iL (0 hours) and at different time points following treatment with 4-OHT.

(H) qRT-PCR analysis of naive markers (*Esrrb*, *Klf4*, *Klf5*, *Sox2*, *Tfcp2l1*, *Rex1*, and *Oct4*) in female and male *Nanog*$^{flox/-}$, Rosa26-CreERT2 ESCs in 2iL (0 hours) and at different time points following treatment with 4-OHT. Data shown are the mean of 3 technical replicates. Error bars represent ± SD.

(I) PCR-based gender determination of the C6 ESC line (XX3).

(J) Western blot analysis of p-STAT3 in female (C6 – XX3) and male (E14tg2a – XY1) ESCs in 2iL, 2i or after 3 days in 1 µM JAKi + 2i.

(K) qRT-PCR analysis of the STAT3-target *Socs3* and naive markers (*Nanog*, *Oct4*, and *Sox2*) in female (C6 – XX3) and male (E14tg2a – XY1) ESCs in 2iL, 2i or after 3 and 5 days in 1 µM JAKi + 2i. Data shown are the mean of 3 technical replicates. Error bars represent ± SD.

(L) PCR-based gender determination of the *Oct4*$^{flox/-}$, Rosa26-CreERT2 ESC lines.

(M) Western blot analysis of OCT4 in female and male *Oct4*$^{flox/-}$, Rosa26-CreERT2 ESCs in 2iL (0 hours) and at different time points following treatment with 4-OHT.

(N) qRT-PCR analysis of naive markers (*Nanog*, *Klf2*, and *Rex1*) in female and male *Oct4*$^{flox/-}$, Rosa26-CreERT2 ESCs in 2iL (0 hours) and at different time points following treatment with 4-OHT. Data shown are the mean of 3 technical replicates. Error bars represent ± SD.

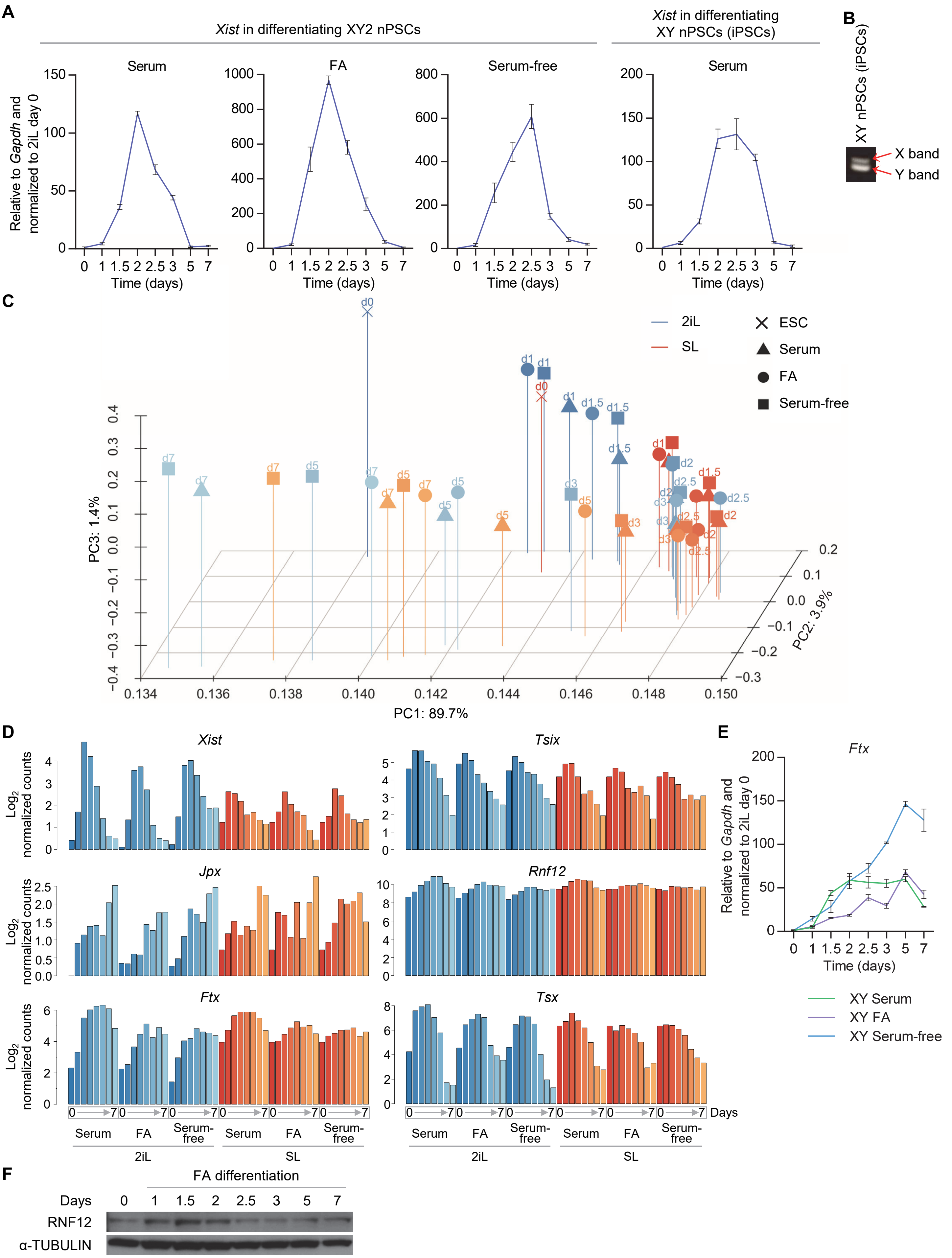

A
Xist in differentiating XY2 nPSCs
Xist in differentiating XY nPSCs (iPSCs)
Serum
FA
Serum-free
Serum
Relative to Gapdh and normalized to 2iL day 0
Time (days)
B
XY nPSCs (iPSCs)
X band
Y band
C
2iL
SL
ESC
Serum
FA
Serum-free
PC3: 1.4%
PC1: 89.7%
PC2: 3.9%
D
Xist
Tsix
Jpx
Rnf12
Ftx
Tsx
Log2 normalized counts
Days
Serum
FA
Serum-free
2iL
SL
E
Ftx
Relative to Gapdh and normalized to 2iL day 0
Time (days)
XY Serum
XY FA
XY Serum-free
F
FA differentiation
Days
0
1
1.5
2
2.5
3
5
7
RNF12
α-TUBULIN

Figure S2. *Xist* is transiently and rapidly upregulated in male nPSC differentiation, Related to Figure 2.

(A) qRT-PCR analysis of *Xist* during differentiation of male (EFC – XY2) ESCs using different conditions, and during embryoid body differentiation of male iPSCs derived from *Rex1*-dGFP neural stem cells. Before differentiation, cells were maintained in 2iL. Data shown are the mean of 3 technical replicates. Error bars represent ± SD.

(B) PCR-based gender determination of the iPSC line.

(C) PCA of differentiating male (E14tg2a – XY1) cells based on differential expression of genes that had an expression of at least Log_2 (normalized counts) 4.5 in at least one of the samples. Before differentiation, ESCs were maintained in 2iL or SL media, as indicated. Data was obtained by strand-specific RNA-seq.

(D) Expression levels are shown during differentiation of male (E14tg2a – XY1) ESCs for *Xist* and other elements of the XIC that have been implicated in the regulation of *Xist*. Before differentiation, ESCs were maintained in 2iL or SL media, as indicated. Data were obtained by strand-specific RNA-seq. Scale represents Log_2 transformed expression value.

(E) qRT-PCR analysis of *Ftx* during differentiation of male (E14tg2a – XY1) ESCs using different conditions. Before differentiation, cells were maintained in 2iL. Data shown are the mean of 3 technical replicates. Error bars represent ± SD.

(F) Western blot analysis of RNF12 during differentiation of male (E14tg2a – XY1) ESCs in FA. Before differentiation, cells were maintained in 2iL.

A

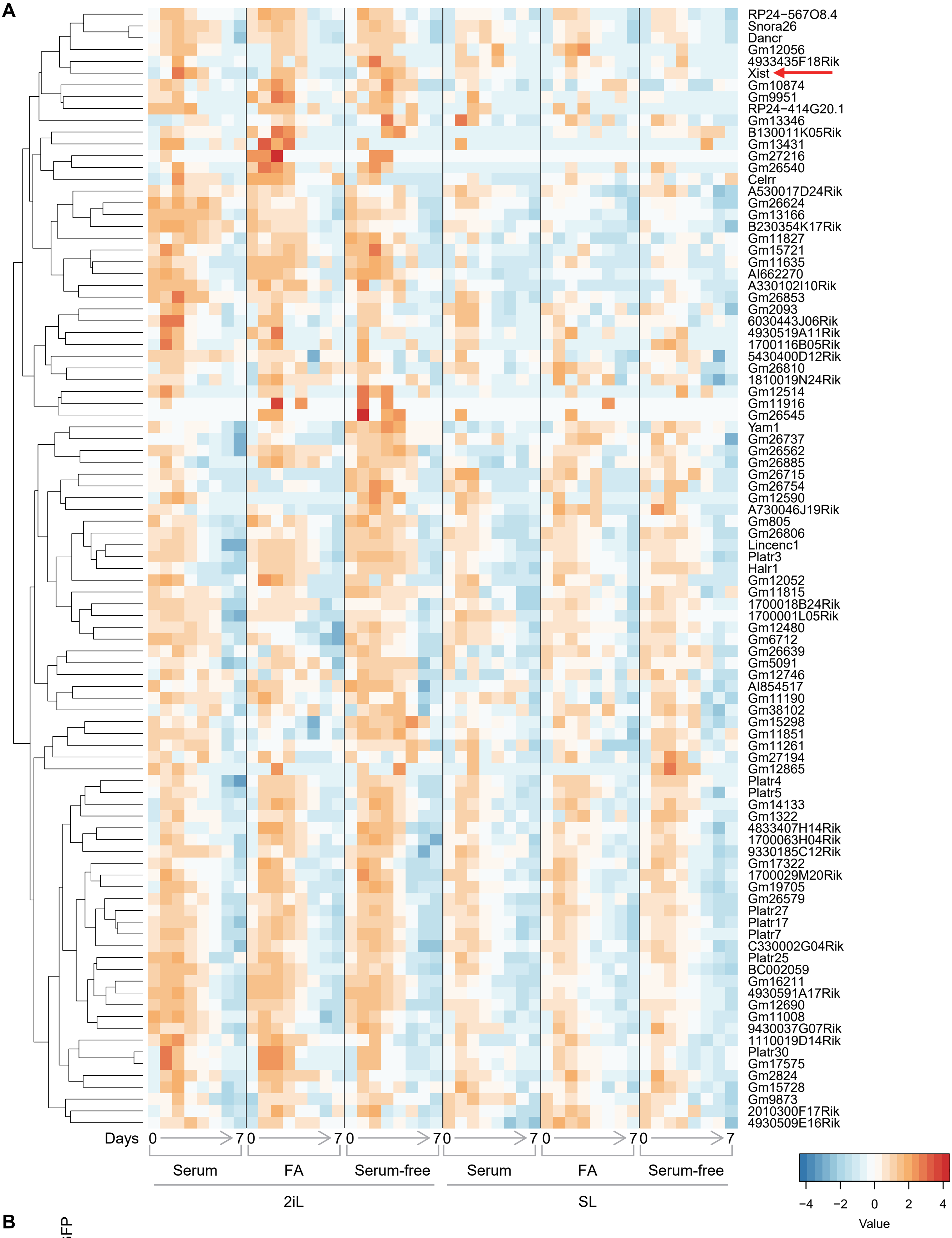
RP24−567O8.4
Snora26
Dancr
Gm12056
4933435F18Rik
Xist
Gm10874
Gm9951
RP24−414G20.1
Gm13346
B130011K05Rik
Gm13431
Gm27216
Gm26540
Celrr
A530017D24Rik
Gm26624
Gm13166
B230354K17Rik
Gm11827
Gm15721
Gm11635
AI662270
A330102I10Rik
Gm26853
Gm2093
6030443J06Rik
4930519A11Rik
1700116B05Rik
5430400D12Rik
Gm26810
1810019N24Rik
Gm12514
Gm11916
Gm26545
Yam1
Gm26737
Gm26562
Gm26885
Gm26715
Gm26754
Gm12590
A730046J19Rik
Gm805
Gm26806
Lincenc1
Platr3
Halr1
Gm12052
Gm11815
1700018B24Rik
1700001L05Rik
Gm12480
Gm6712
Gm26639
Gm5091
Gm12746
AI854517
Gm11190
Gm38102
Gm15298
Gm11851
Gm11261
Gm27194
Gm12865
Platr4
Platr5
Gm14133
Gm1322
4833407H14Rik
1700063H04Rik
9330185C12Rik
Gm17322
1700029M20Rik
Gm19705
Gm26579
Platr27
Platr17
Platr7
C330002G04Rik
Platr25
BC002059
Gm16211
4930591A17Rik
Gm12690
Gm11008
9430037G07Rik
1110019D14Rik
Platr30
Gm17575
Gm2824
Gm15728
Gm9873
2010300F17Rik
4930509E16Rik
Days 0 7 0 7 0 7 0 7 0 7 0 7
Serum
FA
Serum-free
Serum
FA
Serum-free
2iL
SL
−4 −2 0 2 4
Value

B

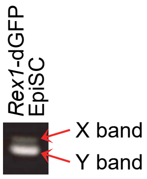
Rex1-dGFP EpiSC
X band
Y band

Figure S3. Expression pattern of long non-coding RNAs in differentiating male nPSCs, Related to Figure 2.

(A) Heatmap depicting the expression profile of long non-coding RNAs during differentiation of male (E14tg2a – XY1) ESCs. Before differentiation, ESCs were maintained in 2iL or SL media, as indicated. Data was obtained by strand-specific RNA-seq. Scale represents z-scores of Log_2 transformed expression values. *Xist* is indicated with a red arrow.

(B) PCR-based gender determination of the *Rex1*-dGFP EpiSC line.

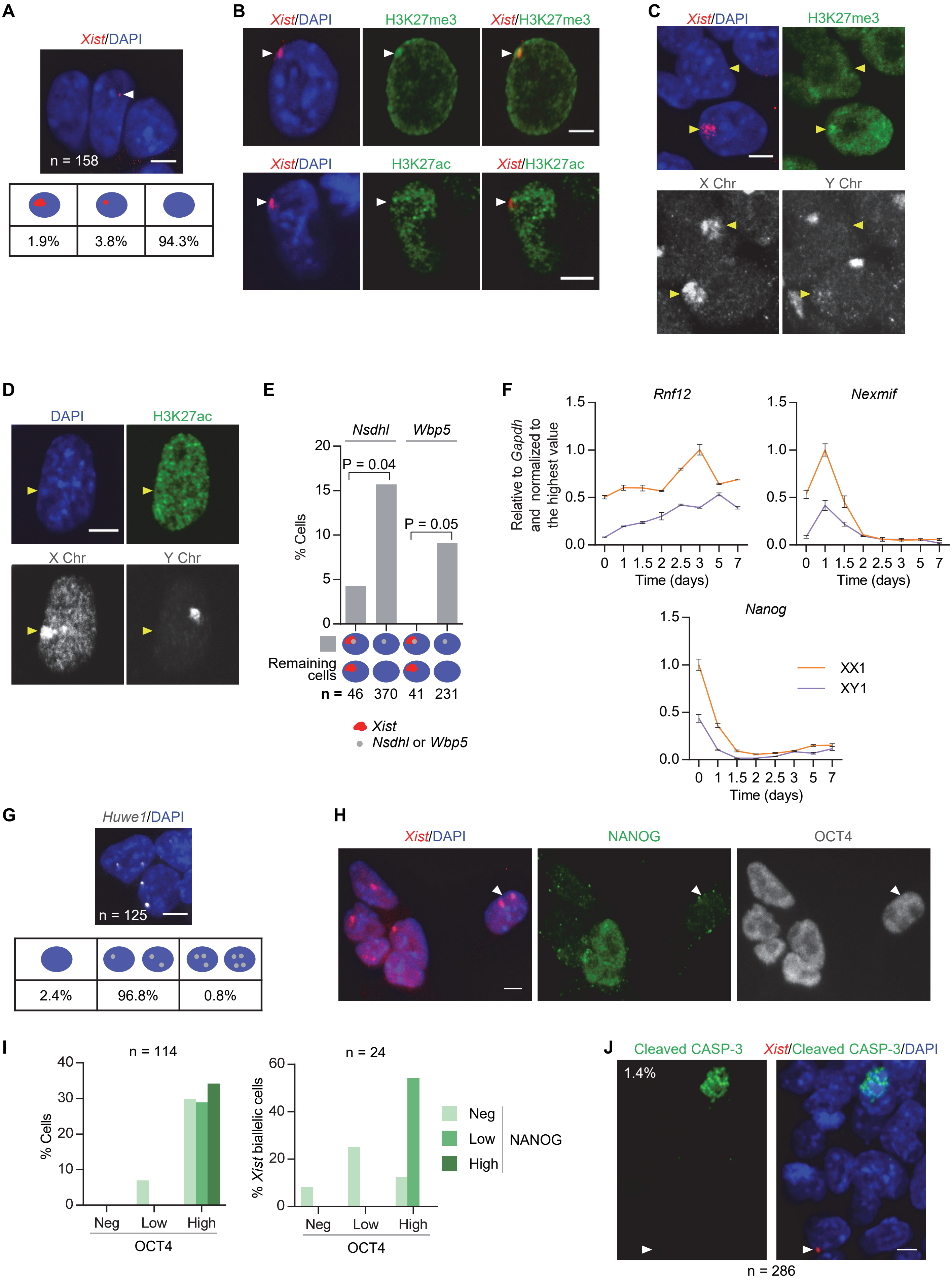

A
Xist/DAPI
n = 158
1.9%
3.8%
94.3%
B
Xist/DAPI
H3K27me3
Xist/H3K27me3
Xist/DAPI
H3K27ac
Xist/H3K27ac
C
Xist/DAPI
H3K27me3
X Chr
Y Chr
D
DAPI
H3K27ac
X Chr
Y Chr
E
Nsdhl
Wbp5
% Cells
P = 0.04
P = 0.05
Remaining cells
n = 46 370 41 231
Xist
Nsdhl or Wbp5
F
Relative to Gapdh and normalized to the highest value
Rnf12
Nexmif
Nanog
Time (days)
XX1
XY1
G
Huwe1/DAPI
n = 125
2.4%
96.8%
0.8%
H
Xist/DAPI
NANOG
OCT4
I
n = 114
% Cells
n = 24
% Xist biallelic cells
Neg
Low
High
OCT4
NANOG
J
Cleaved CASP-3
Xist/Cleaved CASP-3/DAPI
1.4%
n = 286

Figure S4. Males undergo transient XCI, Related to Figure 3.

(A) RNA FISH for *Xist* (red) in male 2iL ESCs at 1.5 days of differentiation in FA using strand-specific probe. Nuclei are shown in blue (DAPI staining). White arrowhead indicates the location of the *Xist* signal. Quantification of the different *Xist* RNA patterns is shown.

(B) Immuno RNA FISH for *Xist* (red) and H3K27me3 or H3K27ac (green) in male 2iL ESCs at 1.5 days of differentiation in FA. Nuclei are shown in blue (DAPI staining). White arrowheads indicate the location of the *Xist* cloud.

(C) Immuno RNA FISH for *Xist* (red) and H3K27me3 (green) in male 2iL ESCs at 1.5 days of differentiation in FA with subsequent DNA FISH XY paint (grayscale). Nuclei are shown in blue (DAPI staining). Yellow arrowheads indicate the location of the X chromosome. A cell lacking H3K27me3 on the X chromosome is shown as a negative control.

(D) Immunofluorescence for H3K27ac (green) in male 2iL ESCs at 1.5 days of differentiation in FA with subsequent DNA FISH XY paint (grayscale). Nuclei are shown in blue (DAPI staining). Yellow arrowheads indicate the location of the X chromosome. A cell showing existence of H3K27ac on the X chromosome is shown as a negative control.

(E) Quantification of the RNA FISH patterns for the X-linked genes *Nsdhl* or *Wbp5* and *Xist* in male 2iL ESCs at 1.5 days of differentiation in FA. Fisher's exact test was used for statistical analysis.

(F) qRT-PCR analysis of *Rnf12*, *Nexmif*, and *Nanog* during differentiation in FA of female (LF1 – XX1) and male (E14tg2a – XY1) ESCs. Before differentiation, cells were maintained in 2iL. Data shown are the mean of 3 technical replicates. Error bars represent ± SD.

(G) RNA FISH in female (LF1) 2iL ESCs with a strand-specific probe detecting X-encoded nascent RNA of *Huwe1* (grayscale). Nuclei are shown in blue (DAPI staining). Quantification of the different RNA FISH patterns is shown.

(H) Immuno RNA FISH for *Xist* (red), NANOG (green) and OCT4 (greyscale) in female (LF1 – XX1) 2iL ESCs at 1.5 days of differentiation in FA. Nuclei are shown in blue (DAPI staining). White arrowhead indicates the location of a cell with biallelic *Xist* upregulation.

(I) Quantification of *Nanog* and *Oct4* expression profile in the bulk population (left) and in cells with biallelic *Xist* expression (right) as shown in (B).

(J) Immuno RNA FISH for *Xist* (red) and cleaved CASPASE-3 (green) in male (E14tg2a) 2iL ESCs at 1.5 days of differentiation in FA. Nuclei are shown in blue (DAPI staining). White arrowhead indicates the location of an *Xist* RNA cloud. The percentage of cleaved CASPASE-3-positive cells is indicated. None of the cells analyzed were positive for both *Xist* and cleaved CASPASE-3.

The scale bar represents 5 μm.
